# Influence of the pH Synthesis of Fe_3_O_4_ Magnetic Nanoparticles on Their Applicability for Magnetic Hyperthermia: An In Vitro Analysis

**DOI:** 10.3390/pharmaceutics17070844

**Published:** 2025-06-27

**Authors:** Bárbara Costa, Eurico Pereira, Vital C. Ferreira-Filho, Ana Salomé Pires, Laura C. J. Pereira, Paula I. P. Soares, Maria Filomena Botelho, Fernando Mendes, Manuel P. F. Graça, Sílvia Soreto Teixeira

**Affiliations:** 1 i3N, Physics Department, University of Aveiro, 3810-193 Aveiro, Portugal; barbaracostaa@ua.pt (B.C.); mpfg@ua.pt (M.P.F.G.); 2 Coimbra Institute for Clinical and Biomedical Research (iCBR) Area of Environment Genetics and Oncobiology (CIMAGO), Institute of Biophysics, Faculty of Medicine, Universidade de Coimbra, Pólo III—Pólo das Ciências da Saúde, Azinhaga de Santa Comba, 3000-548 Coimbra, Portugal; epereira@uc.pt (E.P.); pireslourenco@uc.pt (A.S.P.); mfbotelho@fmed.uc.pt (M.F.B.); fjmendes@estesc.ipc.pt (F.M.); 3 Center for Innovative Biomedicine and Biotechnology (CIBB), Universidade de Coimbra, Rua Larga, 3004-504 Coimbra, Portugal; 4 Clinical Academic Center of Coimbra (CACC), Praceta Professor Mota Pinto, 3004-561 Coimbra, Portugal; 5 Centro de Ciências e Tecnologias Nucleares, C^2^TN, DECN, Instituto Superior Técnico, University of Lisbon, 2695-066 Bobadela, Portugal; vital.filho@ctn.tecnico.ulisboa.pt (V.C.F.-F.); lpereira@ctn.tecnico.ulisboa.pt (L.C.J.P.); 6 i3N/CENIMAT, Department of Materials Science, School of Science and Technology, Nova University of Lisbon, 2829-516 Caparica, Portugal; pi.soares@fct.unl.pt; 7 Coimbra Health School (ESTeSC), Polytechnique University of Coimbra, Rua 5 de Outubro, São Martinho do Bispo, 3045-043 Coimbra, Portugal; 8 H&TRC—Health and Technology Research Center, Coimbra Health Scholl, Polytechnique University of Coimbra, 3046-854 Coimbra, Portugal; 9 European Association of Biomedical Scientists, 1000 Brussels, Belgium

**Keywords:** hydrothermal synthesis, magnetic hyperthermia, magnetite, NaOH, SAR

## Abstract

Nanotechnology, specifically magnetic nanoparticles (MNPs), is revolutionizing cancer treatment. Magnetic hyperthermia is a treatment that, using MNPs, can selectively kill cancer cells without causing damage to the surrounding tissues. **Background/Objectives**: This work aimed to analyze how the synthesis conditions, namely, how the pH of the reaction can influence the magnetic properties of Fe_3_O_4_ nanoparticles for magnetic hyperthermia, using the hydrothermal synthesis. **Methods**: For the hydrothermal synthesis, FeCl_3_·6H_2_O and FeCl_2_·4H_2_O were mixed with different quantities of NaOH to adjust the pH. After obtaining a black precipitate, the samples were placed in an autoclave at 200 °C for 60 h, followed by a washing and drying phase. The obtained MNPs were analyzed using X-Ray Diffraction (XRD), Transmission Electron Microscopy, a Superconducting Quantum Interference Device, Specific Absorption Rate analysis, and cytotoxicity assays. **Results**: Different MNPs were analyzed (9.06 < pH < 12.75). The XRD results showed the presence of various iron oxide phases (magnetite, maghemite, and hematite), resulting from the oxidization of the iron phases present in the autoclave. In terms of the average particle size, it was verified that, by increasing the pH value, the size decreases (from 53.53 nm to 9.49 nm). Additionally, MNPs possess a superparamagnetic behaviour with high SAR values (above 69.3 W/g). **Conclusions**: It was found that the pH of the reaction can influence the size, morphology, magnetization, and thermal efficiency of the MNP. The MNP with the highest composition of Fe_3_O_4_ was synthesized with a pH of 12.75, with a cubic morphology and a SAR value of 92.7 ± 3.2 W/g.

## 1. Introduction

According to ISO 80004-1:2023, magnetic nanoparticles (MNPs) are defined with at least one external dimension within the range of 1–100 nm [1]. Their high surface volume-to-ratio and reduced size are the main contributors to their chemical reactivity [2,3]. Owing to their remarkable magnetic properties, MNPs have found application in various fields, particularly in biomedical sciences [4]. From diagnostics to therapy, their versatility is revolutionizing the use of nanotechnology in biomedicine. More recently, MNPs have gained increasing attention as a promising and efficient approach for cancer treatment.

Depending on the intended application and required properties, MNPs can be synthesized through different methods (chemical, physical, or biological). Among these, the most common ones used to synthesize tailored MNPs include microemulsion, sol–gel, thermal decomposition, co-precipitation, and hydrothermal synthesis [5,6]. In this process, iron precursors are dissolved in aqueous solutions and heated in a Teflon-lined autoclave at high temperatures (120–150 °C) and pressures (1–10 MPa) [7,8,9,10]. Initially, hydrolysis of the iron salts occurs, producing hydroxide intermediates [11], which, upon dehydration, yield the desired MNPs [11,12].

First introduced by Gilchrist et al. in 1957 [13], magnetic hyperthermia (MH) is a therapeutic technique that uses heat to selectively destroy tumour cells [14,15]. The concept of intracellular MH was later presented by Gordon et al. [16] in 1979. This therapy involves directing MNPs to the tumour site, where, under exposure to an external alternating magnetic field (AMF), they generate heat through Néel and Brownian relaxations, raising the local temperature to approximately 42–46 °C. This temperature increase induces physiological changes in tumour cells, triggering apoptotic pathways and ultimately leading to cell death [17]. MH has emerged as a promising cancer treatment strategy, offering the potential to target deep-seated tumours while minimizing damage to the surrounding healthy tissues. Among various types of MNPs, such as ferrites—particularly magnetite (Fe_3_O_4_) and maghemite (γ-Fe_2_O_3_)—are preferred candidates for MH applications [18].

The efficiency of the MH treatment strongly depends on the MNP’s ability to generate heat. From magnetic to morphological properties, different parameters can directly influence the efficiency of the MH treatment. Therefore, controlling the size, shape, and composition of the MNP is essential, making the synthesis method a critical factor in determining MNP performance in MH applications [19]. A study conducted by Rafie et al. [20] emphasized the effect of the pH value on the synthesis process, studying the pH-induced changes in the MNP on different properties, such as the zeta potential, saturation magnetization values, and size distribution. This study synthesized Fe_3_O_4_ MNPs at different pH values (pH 10, pH 11, and pH 12) using the hydrothermal synthesis. The obtained results highlighted that the MNPs synthesized with pH 11 demonstrate a superior performance, with a smaller size, an adsorption capacity of 188.68 mg/g, and higher saturation magnetization values [20]. In addition to this study conducted by Rafie et al., the influence of the pH synthesis on the properties of the MNPs is still uncertain and, therefore, it is essential to perform a more detailed study, focussed on studying how the pH synthesis value can influence the structure, morphology, magnetic properties, and cytotoxicity of these MNPs.

MH using Fe_3_O_4_ nanoparticles has already been approved by the FDA and European authorities for clinical use as an adjuvant therapy for recurrent glioblastoma. It is also being explored as a treatment for other cancers, including lung [21], liver [22], prostate [23], pancreas [24], breast [25], and brain tumours [26].

In their foundational study, Gilchrist et al. demonstrated an ex vivo heating experiment using Fe_3_O_4_ MNPs in canine lymph nodes, showing that as little as 5 mg of nanoparticles could induce a significant temperature rise within 3 min [13]. In vivo studies, such as the one by Rego et al. [27], have also shown encouraging results. In their experiment, Fe_3_O_4_ MNPs coated with aminosilane were used for MH in glioblastoma-induced male Wistar rats. The therapy was administered 21 days after the tumour induction using C6 cells, with an AMF exposure (200 Gauss, 874 kHz) applied for 40 min. A 32.8% reduction in tumour mass was observed compared to the control group [27], reinforcing MH’s potential as a powerful tool for tumour volume reduction [28] and, ultimately, tumour eradication [29].

Hydrothermal synthesis of Fe_3_O_4_ has been widely studied due to its advantages [30,31]. This method allows for precise tailoring of nanoparticle properties [30] by adjusting the synthesis conditions, and high crystalline nanoparticles with controlled size, morphology, and structure [30,32,33]. For example, Haw, C.Y. et al. [33] synthesized spherical superparamagnetic nanoparticles with average diameters of 17.12 nm and a saturation magnetization value of 57.40 emu.g^−1^. Wang, J. et al. [34] obtained highly Fe_3_O_4_ nanorods with varying sizes and high saturation magnetization values (Ms ~ 82.6 emu.g^−1^) via a hydrothermal method.

In the present study, hydrothermal synthesis was employed to produce Fe_3_O_4_ MNPs. The objective was to investigate how variations in synthesis pH influence the average particle size, crystallinity, magnetic properties, and cytotoxicity of the resulting nanoparticles. Five different samples (with pH ranging from 9 to 13) were synthesized and subsequently characterized structurally (via X-Ray diffraction, XRD), morphologically (via transmission electron microscopy, TEM), and magnetically (via SQUID magnetometry and specific absorption rate, SAR, measurements). This study aims to be a highly significant scientific contribution to the field of research into the influence of synthesis pH on the properties of nanoparticles, thereby filling the currently existing gaps in this area.

## 2. Materials and Methods

This work aimed to evaluate how the synthesis conditions could influence the properties of the synthesized MNPs.

### 2.1. Chemical Reagents and Materials

Iron oxide particles were prepared by the hydrothermal method, using iron (III) chloride hexahydrate (FeCl_3_·6H_2_O, ≥99.5%, Merck, Darmstadt, Germany) and iron (II) chloride tetrahydrate (FeCl_2_·4H_2_O, ≥98%, Aldrich, Sigma-Aldrich, Munich, Germany) as precursor reagents. The synthesis pH, from 9 < pH < 13, was adjusted using sodium hydroxide (NaOH, 8N, DBH, Berlin, Germany) as a base, using a LabArt Consort multi-parameter analyser.

Vero cell line (monkey renal epithelial cells) from ATCC CCL-81 (ATCC, Manassas, VA, USA) was used for the cytotoxicity analysis. The cells were seeded in 96-well plates and cultured in Minimum Essential Medium (MEM) purchased from Sigma (Munich, Germany) and supplemented with sodium bicarbonate (Sodium Hydrogen Carbonate, ≥99.7%) from PanReac AppliChem ITW Reagents (PanReac AppliChem, Darmstadt, Germany), antibiotic (Antibiotic Antimycotic Solution), sodium pyruvate (CH_3_COCOONa), and fetal bovine serum (FBS) were purchased from Sigma-Aldrich. Additionally, two colorimetric assays using 3-(4,5-Dimethyl-2-thiazolyl)-2,5-diphenyl-2H-tetrazolium bromide, Thiazolyl blue (MTT, C_18_H_16_BrN_5_S), and Sulforhodamine B (SRB, C_27_H_29_N_2_NaO_7_S_2_) were purchased from Sigma-Aldrich.

### 2.2. Synthesis of Magnetic Nanoparticles Using the Hydrothermal Method

For the synthesis of Fe_3_O_4_ nanoparticles, ferric chloride hexahydrate (FeCl_3_·6H_2_O) and ferrous chloride tetrahydrate (FeCl_2_·4H_2_O) were used as precursors in a molar ratio of 2:1 (Fe^3+^:Fe^2+^). Initially, each precursor was dissolved separately in deionised water. The resulting solutions were then combined and stirred using a magnetic stirrer. To adjust the pH of the reaction medium, an alkaline solution of sodium hydroxide (NaOH) was added dropwise to the iron salt solution under continuous stirring. Upon the formation of a black precipitate, the mixture was transferred to a 100 mL Teflon-lined stainless steel autoclave. The autoclave was sealed and placed in an oven at 200 °C for 60 h. Following the hydrothermal reaction, the resulting precipitate was washed thoroughly with deionised water and ethanol to remove residual ions, and subsequently dried at 60 °C for 24 h.

**Figure 1:**
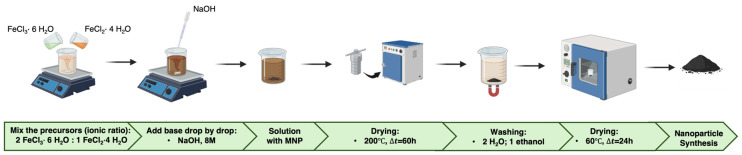
Schematic representation of the hydrothermal synthesis process for iron oxide powders.

Given that the main objective of this study was to assess the influence of synthesis conditions—specifically pH—on the magnetic properties of the nanoparticles, different amounts of NaOH were introduced into the precursor solution. As a result, five distinct samples were synthesized under varying pH conditions: pH 9.06, pH 10.09, pH 11.07, pH 12.12, and pH 12.75. A schematic representation of the hydrothermal synthesis process is presented in Figure 1.

### 2.3. Structural Characterization of the Samples

The MNP structure was investigated using X-ray diffraction (XRD). XRD analysis of the samples was performed using the Panalytical equipment Aeris from Malvern Panalytical (Westborough, MA, USA), using CuKα radiation, with a wavelength of 1.54060 Å, with 2θ angles ranging from 5° to 70°, operating at 15 mA and 40 kV. The Joint Committee for Powder Diffraction Standards—International Center for Diffraction Data (JCPDS) database, which is part of the X’Pert HighScore Plus Panalytical software, version 4.9, was used to identify the crystalline phases.

### 2.4. Morphological Characterization of the Samples

Sample morphology was investigated using Transmission Electron Microscopy (TEM). Therefore, the FEI-Tecnai G2 Spirit Biotwin microscope (Hillsboro, OR, USA) was used. A sample preparation was required, where samples were suspended in deionized water with an ultrasonic probe to prevent agglomeration. A drop of each suspended sample solution was placed on a carbon-coated copper grid. After obtaining a set of representative images for each sample using the Soft Imaging System MegaView III, ImageJ 1.52v was used to determine the average grain size.

### 2.5. Analysis of the Magnetic Behaviour and Thermal Efficiency of the Samples

SQUID magnetometry was used to assess the magnetic properties of the synthesized samples. The magnetization-field (M-H) graphs were obtained using an S700X SQUID magnetometer (Cryogenic Ltd., London, UK). The data was collected for different temperatures (10 K and 300 K) within a magnetic field range from −5 T to 5 T, to obtain the hysteresis curve for each sample.

Regarding thermal efficiency, the Specific Absorption Rate (SAR) analysis quantifies the efficiency of the MNPs in terms of thermal efficiency. Therefore, the D5 series from nB nanoscale Biomagnetics (Zaragoza, Spain) was used to obtain the SAR values. The samples were subjected to an alternating magnetic field (AMF) with an amplitude of 24 kAm^−1^, with a frequency of 388 kHz for 10 min.

### 2.6. Cytotoxicity Analysis of the Samples

To evaluate the cytotoxicity of each sample, MTT and SRB assays were performed using the extract method according to the ISO 10993 [35].

The MNP extracts were prepared at 20 mg/mL in cell culture media and incubated at 37 °C with 5% CO_2_ in a humidified atmosphere for 24 h under rotation.

Vero cells were seeded in 96-well plates (10,000 cells/well), allowed to adhere, and then treated with the 20 mg/mL extract at several dilutions (100%, 50%, 25%, 12.5%, 6.25%). Forty-eight hours after the treatment, the MTT and SRB assays were performed to evaluate the metabolic activity and protein content, respectively, as previously described [36]. Results are presented as the percentage of protein content or metabolic activity normalized to the control.

Statistical analysis was conducted using GraphPad Prism version 10.2.3 for Windows, GraphPad Software, Boston, MA, USA, www.graphpad.com. Non-parametric tests were applied, namely a Wilcoxon signed-rank test was used for statistical comparison between each group and the threshold value of 70%, according to “ISO 10993-5:2009; Biological evaluation of medical devices—Part 5: Tests for in vitro cytotoxicity”, indicating that a reduction in metabolic activity or protein content below this value indicates cytotoxicity.

## 3. Results and Discussion

This section is subdivided into three different subsections analyzing the synthesized samples. Therefore, the analysis and discussion of the structural, morphological, magnetic, and biological analyses will be described.

### 3.1. Structural Characterization

#### X-Ray Diffraction Results

**Figure 2:**
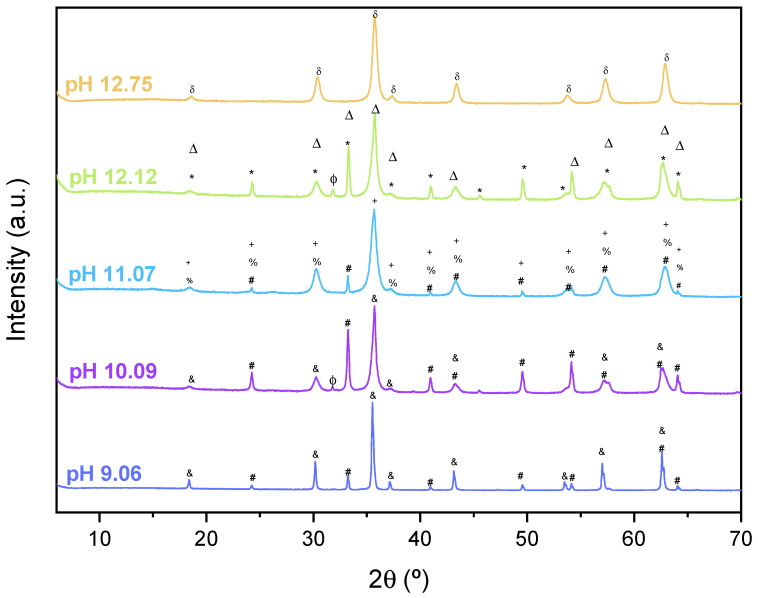
XRD diffractograms were obtained for the synthesized samples using different pH values for the synthesis reaction. (^&^ Fe_3_O_4_ (JCPDS 04-005-404); ^#^ α-Fe_2_O_3_ (JCPDS 01-076-4579); ^ϕ^ NaCl (JCPDS 04-002-1266); ^+^ γ-Fe_2_O_3_ (JCPDS 00-013-7144); ^%^ Fe_2_O_3_ (JCPDS 03-065-0390); * α-Fe_2_O_3_ (JCPDS 00-013-0534); ^Δ^ Fe_3_O_4_ (JCPDS 00-065-0731); ^δ^ Fe_3_O_4_ (JCPDS 01-088-4625).

Figure 2 presents the X-ray diffraction (XRD) patterns of the five synthesized iron oxide samples. Among them, only the sample synthesized at a reaction pH of 12.75 exhibits a pure Fe_3_O_4_ phase composition. In the remaining samples, multiple iron oxide phases were identified: maghemite (indicated in Figure 2 by the * symbol), hematite (identified by the symbols Δ and #), and Fe_2_O_3_ (represented by the % symbol). Additionally, the presence of sodium chloride (NaCl) was confirmed, denoted *ϕ* by the symbol.

Table 1 summarizes the average crystallite size, crystal system, and Goodness of Fit (GoF) values for each of the synthesized samples.

The structural analysis was performed using the Rietveld refinement method, originally described by Hugo Rietveld. This technique applies a full-pattern fitting approach, allowing for precise characterization of crystalline materials by analyzing the entire powder diffraction pattern [37,38]. The method offers significant advantages, particularly in enabling rapid and quantitative evaluation of phase composition [38]. The quality of the refinement is assessed through several parameters, with the Goodness of Fit (GoF) being among the most relevant. The GoF value typically decreases throughout the refinement process, and a value close to 1 indicates a high-quality fit.

The hydrothermal synthesis method is well known for its capacity to produce pure Fe_3_O_4_ phases under a variety of conditions. However, several studies have also reported the use of hydrothermal synthesis to induce phase transitions among different iron oxides [39]. In order to fully understand these transformation processes, it is essential to examine the intermediate phases involved and the specific mechanisms that occur during hydrothermal treatment.

Hydrothermal synthesis is typically carried out in a metallic autoclave. Due to the chemical and crystallographic heterogeneity present on the inner surface of the autoclave, microanodes and microcathodes are formed along the walls [40]. When the system is exposed to an alkaline environment, oxygen evolution occurs at the anode, as described by Equation (1). The excess oxygen generated in this process can act as a key oxidizing agent, partially converting Fe(OH)_2_ and Fe(OH)_3_ into α-FeOOH particles. However, when the molar ratio of Fe^3+^ to Fe^2+^ is maintained at 2:1, a nucleation and growth process of Fe_3_O_4_ particles on the surface of the previously formed α-FeOOH is promoted [40], as illustrated by Equations (2)–(4) [41].

4OH^−^ → O_2_ + 2H_2_O + 4e^−^ (1)

Fe^2+^ + 2 OH^−^ → Fe(OH)_2_ (2)

(3) $$3\mathrm{Fe}(\mathrm{OH}{)}_{2}+\frac{1}{2}O_{2}\;\to \;\mathrm{Fe}(\mathrm{OH}{)}_{2}+2\mathrm{FeOOH}+H_{2}O$$

Fe(OH)_2_ + 2FeOOH → Fe_3_O_4_ + 2H_2_O (4)

(5) $$4\;{\mathrm{Fe}}_{3}O_{4}+O_{2}\;\to \;6\;{\mathrm{Fe}}_{2}O_{3}$$

As shown in Figure 2, the pH value that promotes the formation of a pure Fe_3_O_4_ phase is 12.75. Table 1 summarizes the identified phases, their compositions, crystal systems, and the corresponding Goodness of Fit (GoF) values obtained through Rietveld refinement for each sample. At first glance, it is evident that the Fe_3_O_4_ phase is present in the majority of samples. However, additional iron oxide phases—such as maghemite and hematite—were also detected in most compositions.

Regarding the formation of hematite, it is well documented that in various geological systems, the transformation of magnetite into hematite and maghemite is commonly observed [42]. This transformation is typically attributed to the oxidation of Fe_3_O_4_ by molecular oxygen, as represented in Equation (5). According to Davis et al. [43], maghemite is initially formed through the diffusion of Fe^3+^ ions into the Fe_3_O_4_ structure, followed by further oxidation to hematite. In the current synthesis process, the basic environment favours the partial oxidation of magnetite into maghemite. This occurs as Fe_3_O_4_ particles oxidize to form Fe_2_O_3_ and Fe^2+^. Furthermore, the presence of OH^−^ ions, originating from the NaOH base solution, ensures that the reaction conditions remain strongly alkaline [44].

The presence of NaCl in the samples synthesized at pH 10.09 and pH 12.12 suggests that the post-synthesis washing steps (with water and alcohol) were not sufficient to completely remove the residual NaCl formed during the reaction.

Crystallographically, Fe_3_O_4_ exhibits a typical inverse spinel structure with a cubic $Fd\bar{3}m$ space group, as confirmed by our XRD data and supported by findings from Noh et al. [45]. Hematite, identified in the samples prepared at pH 9.06, 10.09, 11.07, and 12.12, presents a hexagonal structure, consistent with literature reports [46]. The detection of a tetragonal Fe_2_O_3_ phase is indicative of maghemite. Although its crystal structure closely resembles that of magnetite, maghemite contains a defective lattice with approximately one-ninth of the Fe cation sites vacant, which defines its unique crystallographic characteristics [47].

### 3.2. Morphological Characterization

**Figure 3:**
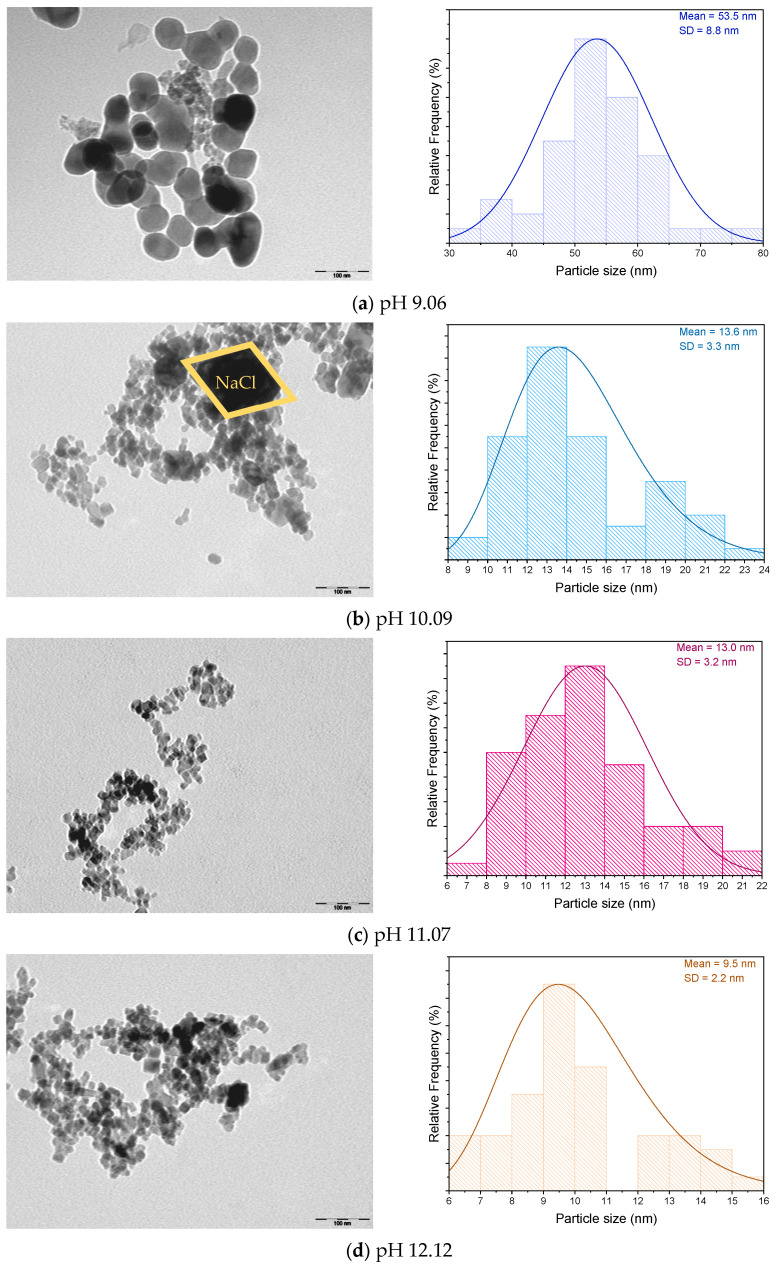

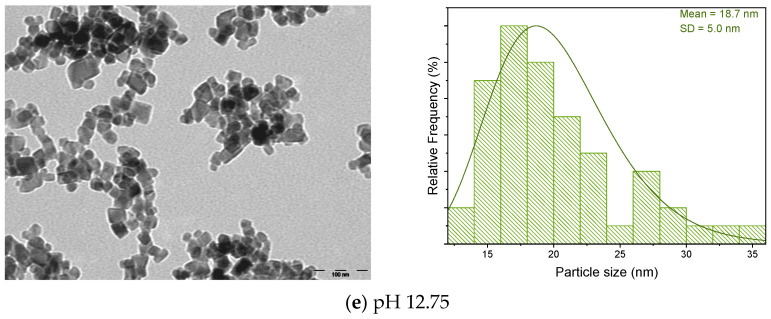
TEM analysis of the obtained MNPs using different pH reaction values: (**a**) pH 9.06; (**b**) 10.09; (**c**) pH 11.07; (**d**) 12.12; (**e**) 12.75.

Transmission Electron Microscopy (TEM) analysis was conducted for each sample to evaluate the influence of the synthesis reaction pH on the size and morphology of the resulting magnetic nanoparticles (MNPs). Figure 3 presents the micrographs along with the corresponding average particle size distribution analysis.

Based on the results presented in Figure 3, it is evident that the increasing the pH value of the synthesis reaction leads to a reduction in the average particle size, decreasing from 53.5 ± 8.9 nm to 9.5 ± 2.2 nm, as previously reported by Rafie, S.F. et al. [20]. However, for the MNPs synthesized at pH 12.75—which displays a pure Fe_3_O_4_ phase—a slight increase in average particle size is observed. This deviation may be attributed to nanoparticle agglomeration, as shown in Figure 3e. Agglomeration can negatively influence magnetic properties by diminishing their performance [48].

Additionally, Figure 3b shows a large particle with a rhombohedral morphology, suggesting the presence of NaCl crystals [49], which aligns with the XRD findings. The formation of NaCl is associated with the synthesis precursors, where Na^+^ ions from the NaOH base combine with Cl^−^ ions from the iron salts.

Regarding morphology, the MNPs synthesized at pH 9.06 exhibit a spherical shape, whereas those synthesized at higher pH values display a more cubic geometry, consistent with findings by Torres-Gómez et al. [7]. These results confirm that the pH of the synthesis reaction directly influences the morphology of the resulting MNPs. Furthermore, it is well established that nanoparticle morphology plays a critical role in determining their magnetic behaviour [50].

### 3.3. Magnetic Characterization

**Figure 4:**
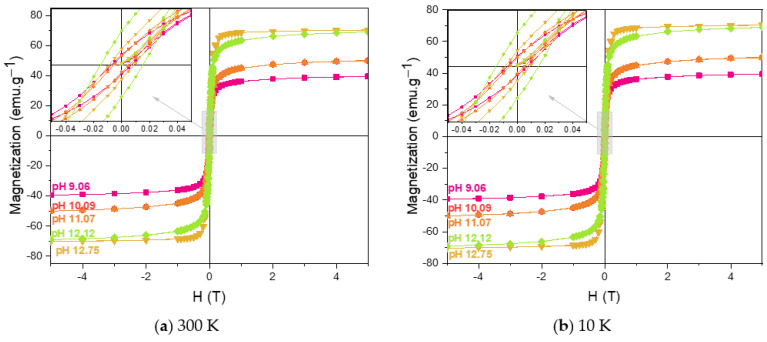
Hysteresis loop obtained for the MNPs synthesized with different pH values at (**a**) 300 K and (**b**) 10 K.

To evaluate the influence of the synthesis reaction pH on the magnetic properties of the synthesized magnetic nanoparticles (MNPs), SQUID magnetometry was employed. The magnetic behaviour was assessed by analyzing the hysteresis loops at both 300 K and 10 K, as shown in Figure 4.

In addition to the magnetic response of MNPs to an external magnetic field, several key parameters are essential for characterizing their magnetic properties, namely saturation magnetization (M_s_), remanent magnetization (M_r_), and coercivity (H_c_). Saturation magnetization (M_s_) refers to the maximum magnetization achieved under an external magnetic field. Remanent magnetization (M_r_) is the residual magnetization that remains after the external field is removed. Coercivity (H_c_) is defined as the intensity of the magnetic field required to reduce the magnetization to zero. Also referred to as the width of the M-H curve, H_c_ is known to be highly dependent on nanoparticle size [51,52].

Table 2 summarizes the values obtained for M_s_, M_r_, and H_c_ at room temperature (300 K) and at 10 K for the various synthesized MNPs. At 300 K, the low H_c_ and M_r_ values indicate that the nanoparticles exhibit a close to superparamagnetic behaviour, similar to that described by Ahmadi, S. et al. [53,54,55]. The non-zero values of H_c_ and M_r_ appear to be primarily associated with the size distribution of the synthesized nanoparticles, as detailed in Figure 3. It is well established that Fe_3_O_4_ nanoparticles with diameters below 20 nm typically exhibit superparamagnetic behaviour [56]. However, Figure 3 shows that, although the average particle size is below 20 nm, the observed size distribution includes some particles larger than 20 nm. This size variation supports the interpretation that the measured H_c_ and M_r_ values may result from the nanoparticle size dispersion. Additionally, M_s_ values were observed to increase with higher pH values during synthesis. This phenomenon can be linked to a decrease in the surface-to-volume ratio and the spin canting effect, which is well-documented in maghemite, magnetite, and hematite nanoparticles [53,57,58,59,60,61]

At 10 K, the M-H curves exhibit more pronounced hysteresis. This is reflected in the higher M_r_ and H_c_ values compared to those measured at 300 K [62]. Moreover, M_s_ values increased across all samples at 10 K, further supporting the temperature dependence of magnetic properties like H_c_, M_r_, and M_s_ in Fe_3_O_4_ nanoparticles [63,64].

At 300 K, the sample synthesized at pH 12.75 displayed an M_s_ value of 70.43 emu.g^−1^, which is lower than the bulk magnetite value of 92 emu.g^−1^ [63]. This reduction is primarily attributed to the smaller particle size. For the other samples, the even lower M_s_ values are likely due to the presence of secondary iron oxide phases such as maghemite (M_s_ ≈ 76 emu.g^−1^ [65]) and hematite (M_s_ ranging from 0.1 to 0.4 emu.g^−1^ [66]), the latter being antiferromagnetic at room temperature, which significantly reduces overall M_s_ values.

**Figure 5:**
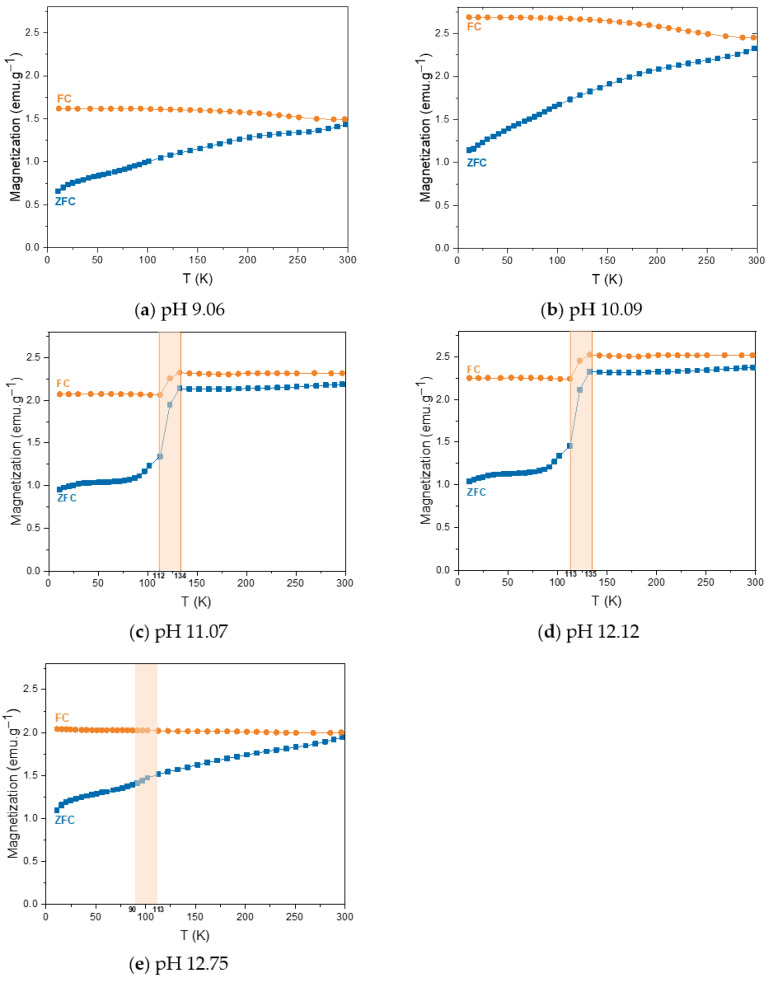
ZFC and FC curves at 50 Oe from 10 to 300 K obtained for the MNPs using different pH reaction values: (**a**) pH 9.06; (**b**) 10.09; (**c**) pH 11.07; (**d**) 12.12; (**e**) 12.75.

Figure 5 depicts the magnetization dependence with temperature in the field cooled (FC) and zero field cooled (ZFC) states, for all the synthesized samples, with an applied field of 50 Oe. In Figure 5c–e, the Verwey transition of magnetite is easily evidenced for samples pH 11.07, 12.12, and 12.75, characteristic of the charge-order transition of the magnetite and observed typically around 122 K, a clear sign of highly crystalline and stoichiometric magnetite [67]. This transition is highly size and shape dependent, and can vary depending on the synthesis method, as well as the crystallinity degree [67,68,69].

From Figure 5 can also be observed that the magnetization as ZFC increases with increasing temperature for all the samples, indicating that the nanoparticles are in a magnetically blocked state, as confirmed by the hysteresis curves presented in Figure 4 [70].

Considering that one of the main objectives of this work is to assess the influence of the pH of the synthesis on the magnetic properties of nanoparticles to be applied in magnetic hyperthermia, it is crucial to evaluate the thermal efficiency of the nanoparticles.

The Specific Absorption Rate (SAR) is the property that quantifies a particular material’s capacity to generate heat. It is used to characterize the thermal efficiency of a magnetic material, being an important parameter to quantify and analyze the suitability of a particular nanoparticle to be used for magnetic hyperthermia. The following equation calculates the SAR:

(6) $$SAR=\frac{m_{l}\times {\;C}_{l}+m_{l}\times {\;C}_{NP}}{m_{Fe}}\times \left(\frac{dT}{dt}\right)$$

In Equation (6), $m_{l}\;$ is the mass of the fluid, $m_{Fe}$ is the iron mass of the solution, ${\;C}_{l}$ and ${\;C}_{NP}$ is the specific heat of the liquid and the magnetic material, respectively, and $\left(\frac{dT}{dt}\right)$ is the variation of temperature within a certain period of time.

**Figure 6:**
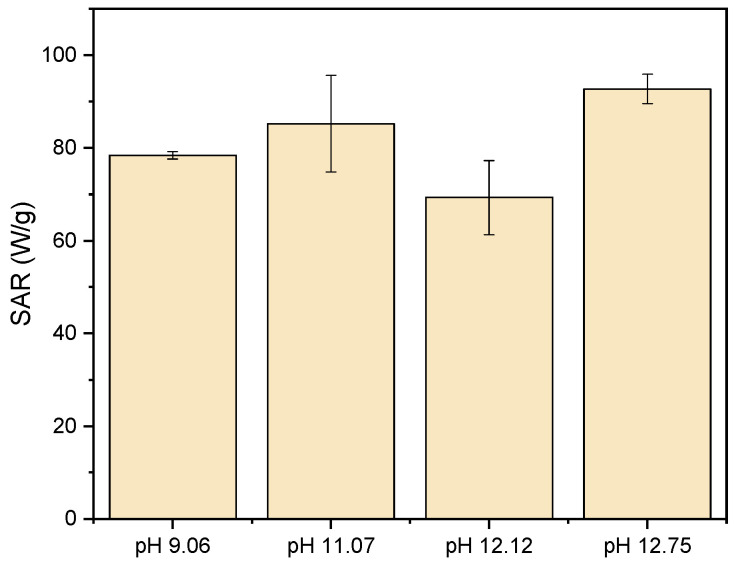
Representation of the SAR values obtained for each synthesized sample at different pH values.

SAR values can be affected by several factors, such as particle size, M_s_, crystallinity, and nanoparticle agglomeration [57,58,59,60,61]. Besides these parameters, it is also important to note that both the frequency and the field amplitude of the AMF are significantly correlated with the obtained SAR value [61]. As stated in the introduction, MNPs generate heat through Brownian and Néel relaxation mechanisms. These processes, which involve the rotation of magnetic moments, can be influenced by magnetic interactions occurring between nanoparticles. Such interactions may alter the efficiency of energy dissipation, thereby affecting the resulting SAR values [71]. Figure 6 represents the obtained SAR values for each of the synthesized sample.

Table 3 represents the obtained SAR values and the respective temperature variation for the different MNPs synthesized using different pH synthesis values. Due to the extreme aggregation that the particles pH 10.09 displayed in the SAR analysis, which led to a very large inaccuracy, the data for sample pH 10.09 is not displayed. In general, the obtained SAR values are lower when compared with those obtained by Lemine, O. et al. for Fe_3_O_4_ particles (SAR ~ 163 W/g for an average particle size of 10–15 nm, with a concentration of 5 mg/mL) [61]. This difference can be attributed to the visible MNP agglomeration, as can be seen in TEM images (Figure 3). The MNPs with the highest SAR were synthesized at a pH value of 12.75. This is due to the composition of a pure phase of Fe_3_O_4_, since hematite and maghemite present lower SAR values than the ones obtained for pure Fe_3_O_4_ [72]. Therefore, regarding magnetic hyperthermia application, the MNPs synthesized at a pH value of 12.75 are in fact the most suitable for its thermal efficiency.

### 3.4. Cytotoxicity Analysis

To assess the cytotoxicity of the synthesized nanoparticle, MTT and SRB assays were performed according to the ISO 10993 “Biological evaluation of medical devices—Part 5: Tests for in vitro cytotoxicity”, using the extract method. While the SRB assay evaluates the protein content, the MTT assay evaluates the metabolic activity. According to the previous ISO, if metabolic activity and protein content decrease for values below 70%, the synthesized MNPs are cytotoxicity [73].

**Figure 7:**
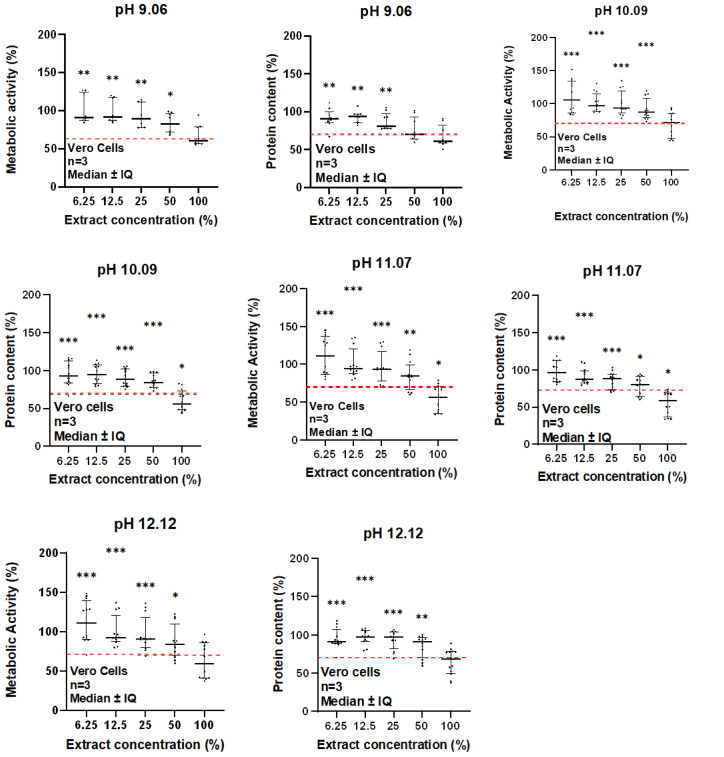
Cytotoxic analysis of the synthesized MNPs using different pH values for the synthesis reaction. (**a**) MTT results; (**b**) SRB results. A Wilcoxon signed-rank test was used for statistical comparison between each group and the threshold value of 70%, according to ISO 10993-5:2009. For each graph, the data is represented as median ± IQ of the percentage of metabolic activity or protein content normalized to control for a non-normal distribution (* *p* ≤ 0.05, ** *p* ≤ 0.01, *** *p* ≤ 0.001).

Figure 7 depicts the obtained results for the MTT and SRB assays. According to the observed results, it is possible to conclude that both MTT and SRB results are consistent and in agreement. In the case of the MTT results, the only sample denoting a significant decrease in the metabolic activity, below the 70% threshold, was the one synthesized at pH 11.07 for the extract concentration of 100%. For the SRB assays, the ones that denoted a significant decrease in protein content below 70% were the MNPs synthesized with a pH of 10.09 and 11.07, for the extract concentration of 100%. Therefore, it seems that up to a concentration of 20 mg/mL, none of the synthesized MNPs exhibit cytotoxic behaviour. Note that for in vitro and in vivo assays, the concentration of Fe_3_O_4_ is usually less than 20 mg/mL [61]. The concentration of Fe_3_O_4_ used for these assays was intentionally higher than the reported one to verify the cells’ response. The cytotoxicity assays were carried out in non-tumoral cell lines to evaluate if these nanoparticles are cytotoxic in a health environment.

## 4. Conclusions

Cancer remains a major public health concern worldwide. The considerable limitations associated with conventional therapies have driven the scientific community to explore alternative and more effective treatment approaches. In this context, nanotechnology—particularly magnetic hyperthermia—has emerged as a promising strategy for cancer therapy. This technique relies on magnetic nanoparticles, which, under the influence of an alternating magnetic field, generate heat via Brownian and Néel relaxation mechanisms. The resulting temperature increase (typically in the range of 42–46 °C) can induce tumour cell death through apoptotic pathways.

For magnetic hyperthermia to be effective, the selection of MNPs with high specific absorption rate values and reduced particle size is essential. The present study aimed to investigate how the synthesis conditions—specifically, the pH of the reaction—affect the physicochemical and magnetic properties of Fe_3_O_4_ nanoparticles synthesized via the hydrothermal method. By varying the amount of NaOH added to the precursor solution, it was observed that increasing the pH promoted nucleation and growth of the Fe_3_O_4_ phase, while simultaneously contributing to a reduction in particle size.

Magnetic characterization revealed that all synthesized samples exhibited a close to superparamagnetic behaviour, accompanied by high SAR values, indicating their potential suitability for hyperthermia applications. The best magnetic performance was obtained for the MNPs synthesized at pH 12.75, with the highest SAR, 92.7 ± 3.2 W/g, and M_s_, 70.43 emu.g^−1^ values. Furthermore, the cytotoxicity assays demonstrated that at concentrations below 20 mg/mL, the nanoparticles did not induce significant toxic effects on non-cancerous cells.

This study reinforces the importance of synthesis parameters—particularly pH—in tailoring the properties of Fe_3_O_4_ nanoparticles via hydrothermal methods. The findings contribute to a broader understanding of how controlled synthesis can optimize magnetic nanoparticle performance for biomedical applications and open avenues for future investigations into additional parameters influencing their functionality.

## Figures and Tables

**Table 1:** Identify the crystalline phases, composition, crystal system, and the obtained Goodness of Fit (GoF) value for each of the synthesized samples.

Sample	Crystalline Phase	Composition	Crystal System	GoF
pH 9.06	Fe_3_O_4_	82%	Cubic	1.99
	α-Fe_2_O_3_	18%	Hexagonal	
pH 10.09	Fe_3_O_4_	52.7%	Cubic	2.05
	α-Fe_2_O_3_	46.3%	Hexagonal	
	NaCl	1%	Cubic	
pH 11.07	γ-Fe_2_O_3_	94.1%	Cubic	2.19
	α-Fe_2_O_3_	5.3%	Hexagonal	
	Fe_2_O_3_	0.6%	Tetragonal	
pH 12.12	Fe_3_O_4_	65.6%	Cubic	2.21
	α-Fe_2_O_3_	32.3%	Hexagonal	
	NaCl	2.1%	Cubic	
pH 12.75	Fe_3_O_4_	100%	Cubic	2.45

**Table 2:** Analysis of the magnetic parameters obtained at 300 K and 10 K using the SQUID (M_s_, M_r_ and H_c_).

Sample	M_s_ (emu.g^−1^)		M_r_ (emu.g^−1^)		H_c_ (mT)	
	300 K	10 K	300 K	10 K	300 K	10 K
pH 9.06	39.46	44.32	3.71	14.38	7	46
pH 10.09	50.24	68.86	3.20	19.69	6	39
pH 11.07	49.79	56.22	3.20	16.81	6	44
pH 12.12	69.03	76.91	12.07	12.65	15	47
pH 12.75	70.43	74.90	5.68	11.14	9	23

**Table 3:** SAR values and temperature variation obtained for each of the synthesized MNPs.

Sample	SAR (W/g)	ΔT (°C)
pH 9.06	78.4 ± 0.8	25.5 ± 0.7
pH 11.07	85.2 ± 10.4	29.9 ± 4.6
pH 12.12	69.3 ± 8.0	21.9 ± 4.3
pH 12.75	92.7 ± 3.2	36.1 ± 1.4

## Data Availability

The data presented in this study are contained within the manuscript.
